# Combinational losses of synucleins reveal their differential requirements for compensating age-dependent alterations in motor behavior and dopamine metabolism

**DOI:** 10.1016/j.neurobiolaging.2016.06.020

**Published:** 2016-10

**Authors:** Natalie Connor-Robson, Owen M. Peters, Steven Millership, Natalia Ninkina, Vladimir L. Buchman

**Affiliations:** a School of Biosciences, Cardiff University, Cardiff, UK; b Institute of Physiologically Active Compounds RAS, Moscow Region, Russian Federation

**Keywords:** Synuclein, Nigrostriatal system, Knockout mice, Null mutant, Parkinson's disease, Dopamine

## Abstract

Synucleins are involved in multiple steps of the neurotransmitter turnover, but the largely normal synaptic function in young adult animals completely lacking synucleins suggests their roles are dispensable for execution of these processes. Instead, they may be utilized for boosting the efficiency of certain molecular mechanisms in presynaptic terminals, with a deficiency of synuclein proteins sensitizing to or exacerbating synaptic malfunction caused by accumulation of mild alterations, which are commonly associated with aging. Although functional redundancy within the family has been reported, it is unclear whether the remaining synucleins can fully compensate for the deficiency of a lost family member or whether some functions are specific for a particular member. We assessed several structural and functional characteristics of the nigrostriatal system of mice lacking members of the synuclein family in every possible combination and demonstrated that stabilization of the striatal dopamine level depends on the presence of α-synuclein and cannot be compensated by other family members, whereas β-synuclein is required for efficient maintenance of animal's balance and coordination in old age.

## 1 Introduction

Among several proteins whose malfunction has been linked to molecular events leading to neuronal dysfunction in Parkinson's disease (PD), α-synuclein deservingly hold the status of a primary culprit due to its causative role in certain familial forms and a pivotal role in the formation of histopathological hallmarks in both familial and idiopathic forms of the disease [reviewed in (Dev et al., 2003, Venda et al., 2010)]. Moreover, polymorphisms within the locus encoding α-synuclein have been found to be associated with the increased risk of PD development (Kay et al., 2008, Mizuta et al., 2008, Pankratz et al., 2009, Scholz et al., 2009, Sutherland et al., 2009), with recent studies suggesting that disease progression to the symptomatic stage correlates with the spreading of pathologic α-synuclein aggregates to dopaminergic neurons of the substantia nigra pars compacta (SNpc) (Braak et al., 2006, Desplats et al., 2009, Luk et al., 2012, Recasens et al., 2014).

Aggregation of α-synuclein not only generates various products intrinsically toxic for neurons but also depletes the pool of functional α-synuclein, particularly in presynaptic terminals, its normal site of localization and function. The importance of α-synuclein for the efficient function of vertebrate presynaptic neurotransmitter storage and release machinery has been demonstrated in experiments with live animals or primary neurons derived from either animals overexpressing various forms α-synuclein or depleted of endogenous α-synuclein by targeted inactivation of *Snca*, gene encoding mouse α-synuclein (Abeliovich et al., 2000, Cabin et al., 2002, Chandra et al., 2004, Chandra et al., 2005, Janezic et al., 2013, Lim et al., 2010, Lin et al., 2012, Nemani et al., 2010, Senior et al., 2008, Taylor et al., 2014, Yavich et al., 2004, Yavich et al., 2005, Yavich et al., 2006).

However, in models with transgenic α-synuclein expression, it is problematic to identify the chain of events leading to alterations of synaptic functions. A modified or overexpressed exogenous α-synuclein may hijack and compromise the endogenous α-synuclein function. Alternatively, functional alterations may be caused by the novel gain-of-function effects of the exogenous α-synuclein, which can be completely unrelated to any of normal functions of the endogenous protein or affect them only indirectly. On the other hand, interpretation of experimental data obtained in α-synuclein null mutant mice is complicated by the expression in vertebrate neurons, including dopaminergic neurons of SNpc, of 2 closely related members of the synuclein family, β-synuclein and γ-synuclein. These 2 proteins are not known to be directly involved in etiology or pathogenesis of PD but can function in the same molecular processes in neuronal synapses as α-synuclein and therefore, compensate, at least partially, for its loss. This functional redundancy might mask consequences of α-synuclein depletion in the SNpc of null mutant mice.

In the absence of the interfamily compensation, that is, following inactivation of all 3 synuclein-encoding genes, animals develop more pronounced behavioral, physiological, and biochemical changes in their nervous system, including the nigrostriatal axis, than animals with null mutations for 1 or 2 of these genes. Importantly, synaptic malfunction develops gradually and its manifestations become detectable only in aging triple synuclein null mutant mice (Anwar et al., 2011, Burre et al., 2010, Greten-Harrison et al., 2010). These observations suggest that the presence of synucleins delays functional decline of neuronal synapses in the aging nervous system. However, the impact of each family member on this process might be different. Indeed, a significant, although not sufficient to cause functional alterations, decrease of striatal dopamine level has been observed only in adult α/β-synuclein double and aged α-synuclein but not in adult α/γ-synuclein double and aged γ-synuclein null mutant mice (Al-Wandi et al., 2010, Chandra et al., 2004, Robertson et al., 2004).

For synucleins, as any other proteins that affect efficiency rather than execution or robust regulation of intracellular processes, it is particularly important to consider effects of external factors when comparing data obtained in different setups. In particular, for studies of mice lacking such proteins, genetic background and environmental conditions might significantly affect experimental data, and at least some discrepancies in results obtained in similar studies of synuclein null mutant mice carried out in different laboratories may have been caused by differences in these parameters.

Therefore, to address the role of each member of the synuclein family in maintaining the efficiency of the nigrostriatal system function in aged animals, we carried out a breeding programme aimed to produce mouse lines lacking 1, 2, or all 3 synucleins and control wild-type mice, on the same C57Bl6/J genetic background, and systematically studied motor behavior and major parameters of the nigrostriatal system in cohorts of aged mice of all 8 genotypes.

## 2 Material and methods

### 2.1 Generation of double and triple synuclein null mutant animals

Generation of α-synuclein, γ-synuclein, and α-synuclein/γ-synuclein double null mutant mice on C57Bl6J (Charles River) background was described previously (Abeliovich et al., 2000, Ninkina et al., 2003, Robertson et al., 2004). Heterozygous β-synuclein null mutant mice (Chandra et al., 2004) on C57Bl6J background were further backcrossed with C57Bl6J (Charles River) mice for 6 generations in the Cardiff University Transgenic Animal Unit before breeding with α-synuclein/γ-synuclein double null mutant mice. Resultant triple heterozygous animals were intercrossed to produce founders of triple null mutant, double null mutant, and wild-type colonies used in this study. Thus, all studied animals were on the same C57Bl6J (Charles River) genetic background. Mice were maintained in conventional open-lid cages with ad libitum access to standard chow and water. Mouse genotyping was carried out as described previously (Chandra et al., 2004, Ninkina et al., 2003, Robertson et al., 2004). Animals were sacrificed by a Schedule 1 method or terminally anesthetized followed by perfusion fixation, and tissues were collected and coded. Individuals that performed all further analyses were blinded to the sample genotype. All animal work was carried out in accordance with the United Kingdom Animals (Scientific Procedures) Act (1986).

### 2.2 Behavioral tests

#### 2.2.1 Inverted grid test

Mice were placed onto a 30 cm by 30 cm square mesh consisting of 5-mm squares of 0.5-mm diameter wire. The grid was slowly rotated to the inverted position and held above a thick layer of bedding material. If a mouse fell from the grid earlier than the maximum test time of 1 minute the latency to fall was noted, and after a 10-minute rest period in the home cage, the test was repeated. The best result from 3 attempts was included in the statistics.

#### 2.2.2 Rotarod test

Mice were trained and then tested for 5 minutes on the accelerating (4–40 rpm) Ugo Basile 7650 rotarod as described previously (Robertson et al., 2004). Each mouse was tested 3 times with at least 30-minute rest period between trials. The mean latency to fall for these 3 trials was included in the final statistics.

### 2.3 Immunohistochemistry and neuronal cell counts

Mouse brains were collected, fixed with Carnoy's fixative, processed, embedded, and cut using an HM 310 microtome (Microm International). Sections of 8 μm thick were mounted onto poly-L-lysine–coated slides followed by staining with antibodies against tyrosine hydroxylase ([TH], mouse monoclonal antibody, clone TH-2, Sigma) diluted 1:1000. For detection, secondary biotinylated anti-mouse or anti-rabbit antibodies, Elite plus kits (Vector laboratories), and 3,3’-diaminobenzidine as chromogen for the peroxidase reaction were used. The borders of the substantia nigra and ventral tegmental area (VTA) on these sections were outlined using distribution atlas of TH-positive cells (Hokfelt et al., 1984). The number of TH-positive neurons was assessed by stereological counting as described in our previous publications (Al-Wandi et al., 2010, Robertson et al., 2004). Briefly, the first section for counting was randomly chosen from the first 10 sections that included SNpc/VTA region. Starting from this section, every TH-positive cell with a clearly seen nucleus was counted on the every fifth section through the whole region. The Axiovision imaging program (Carl Zeiss Vision) was employed to measure diameters of 30 nuclei of dopaminergic neurons in the SNpc of every mouse brain included in this study. The nuclei were chosen randomly and the distance measured as the horizontal length as they appeared on screen. A mean was calculated for each animal and used for Abercrombie's correction (Abercrombie, 1946) to obtain an actual number of TH positive cells in the structure.

### 2.4 High-pressure liquid chromography (HPLC) analysis of striatal neurochemicals

Mouse dorsal striata were dissected, immediately snap-frozen in liquid nitrogen, and kept at −80 °C. After extraction with 0.06-M HClO_4_, concentrations of striatal dopamine, 3,4-dihydroxyphenylacetic acid (DOPAC), and homovanillic acid (HVA) were measured by HPLC with electrochemical detection using a 4.6 × 150-mm Microsorb C18 reverse-phase column (Varian) and Decade II ECD with a Glassy carbon working electrode (Antec Leyden) set at +0.7 V with respect to an Ag/AgCl reference electrode. For measuring dopamine and its metabolites, the mobile phase consisted of 12% methanol (v/v), 0.1-M monosodium phosphate, 2.4-mM 1-octane sulphonic acid, 0.68-mM EDTA, pH 3.1.

### 2.5 Statistical analysis

All data are presented as means ± standard error of the mean. Nonparametric Kruskal–Wallis analysis of variance with post hoc Whitney–Mann *U*-test was used for assessing statistical significance of the difference between groups. The χ^2^ test was used for dichotomous variables (completed or failed the inverted grid test) and Spearman's rank test to reveal correlation between various parameters within groups. Statistical analysis was performed using SPSS/PASW Statistics versions 16.0 or 18.0 (SPSS, Chicago, IL, USA), GraphPad Prism 4.0 (GraphPad Software, San Diego, CA, USA), and GB-Stat PPC 6.5.4 (Dynamic Microsystems, Inc, Silver Spring, MD, USA).

## 3 Results

### 3.1 Mouse welfare and survival rate are not affected by inactivation of synuclein genes in any possible combination

**Fig. 1:**
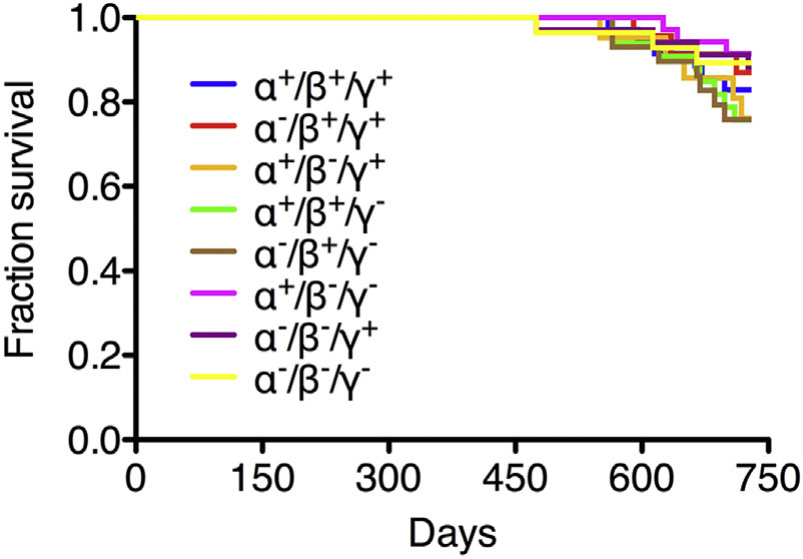
Normal life span of synuclein null mutant mice. Kaplan–Meier survival plot demonstrates survival rate up to the age of 2 years in cohorts of wild-type mice and mice carrying homozygous synuclein null alleles in any of 7 possible combinations. Log rank and generalized Wilcoxon tests for equality of survival did not reveal significant difference between any of studied animal groups (*p* > 0.05). The number of animals in groups: 35 (α^+^β^+^γ^+^), 23 (α^−^β^+^γ^+^), 21 (α^+^β^−^γ^+^), 33 (α^+^β^+^γ^−^), 29 (α^−^β^+^γ^−^), 39 (α^+^β^−^γ^−^), 34 (α^−^β^−^γ^+^), and 28 (α^−^β^−^γ^−^).

Experimental cohorts of 7 synuclein-deficient genotypes and control wild-type male mice were produced on C57Bl6J background as described in Section 2.1. Most animals in all groups survived up to the age of 2 years at which stage they were tested, humanely sacrificed, and tissues collected for histologic and biochemical assessments. No signs of neurologic or other specific health problems were observed in any of the studied cohorts, and some early casualties were caused by random events and not correlated with animals' genotypes. Statistical analysis of the animal life span censored at 2 years demonstrated no difference between groups (Fig. 1).

### 3.2 Compromised motor performance of aged mice lacking β-synuclein

**Fig. 2:**
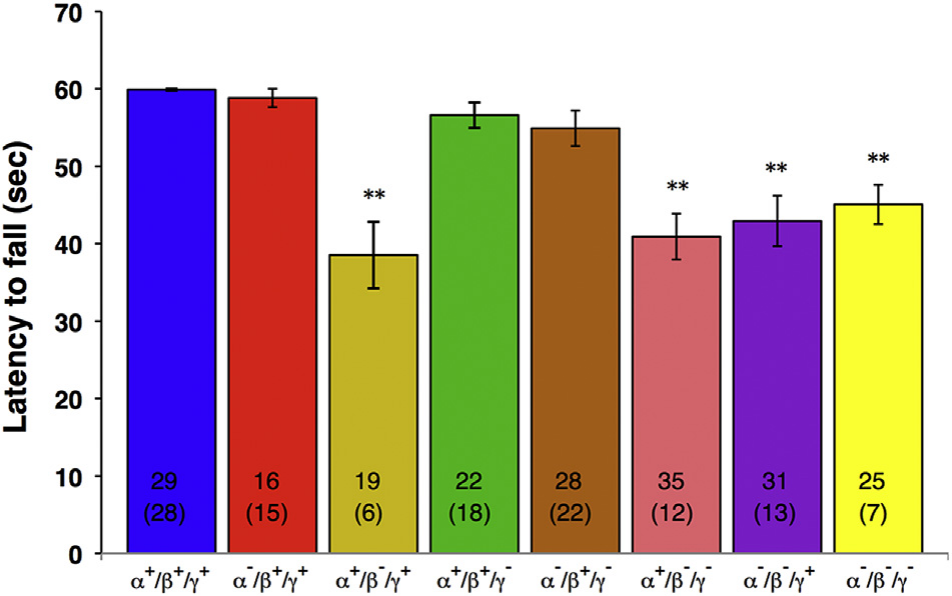
Animal performance on the inverted grid test. Animals were tested as described in Section 2.2.1. The bar chart shows means ± standard error of the mean of latency to fall for groups of 2-year-old wild-type and various synuclein knockout mice. Asterisks denote statistically significant difference between knockout and wild-type groups (^∗∗^*p* < 0.01, Kruskal–Wallis analysis of variance with post hoc Mann–Whitney test). For each group, the number of animals tested and, in parentheses, stayed on the grid for the whole 60-second test in at least 1 of 3 trials is shown at the bottom of the corresponding bar.

**Fig. 3:**
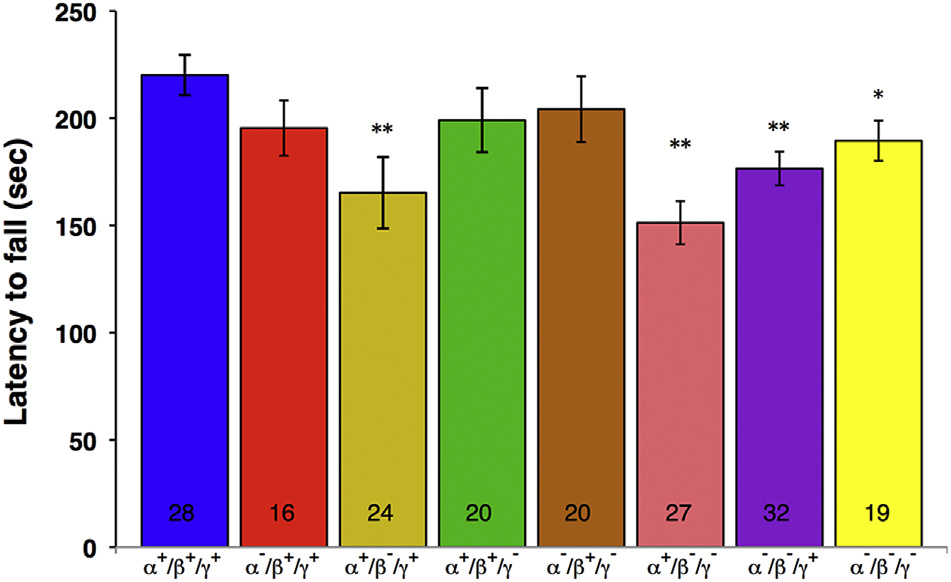
Animal performance on the accelerating rotarod test. Animals were tested as described in Section 2.2.2. The bar chart shows means ± standard error of the mean of latency to fall for groups of 2-year-old wild-type and various synuclein knockout mice. Asterisks denote statistically significant difference between knockout and wild-type groups (^∗^*p* < 0.05, ^∗∗^*p* < 0.01, Kruskal–Wallis analysis of variance with post hoc Mann–Whitney test). The number of animals tested for each group is shown at the bottom of the corresponding bar.

At the age of 2 years, motor performance of synuclein null mutant and control mice was tested in 2 experimental paradigms. The inverted grid test was used to assess coordination and grip strength, with accelerated rotarod test used to evaluate sensorimotor function, coordination, and endurance. Animals lacking β-synuclein, either singularly or in combination with any other member(s) of the family, on average stayed on the inverted grid for a significantly shorter time than wild-type mice, whereas animals expressing β-synuclein were indistinguishable from wild-type mice even when lacking any or all other synucleins (Fig. 2). Moreover, only in groups of mice homozygous for inactivated β-synuclein gene was the success rate at the inverted grid test significantly decreased compared with the wild-type group (Fig. 2, χ^2^ *p* value <0.05). Consistently, mice lacking β-synuclein gene performed significantly worse than wild-type mice in the accelerated rotarod test, whereas the results for mice with intact β-synuclein alleles were not significantly different from the results for wild-type animals (Fig. 3).

### 3.3 Reduced dopamine content in the dorsal striatum of aged mice lacking α-synuclein

**Fig. 4:**
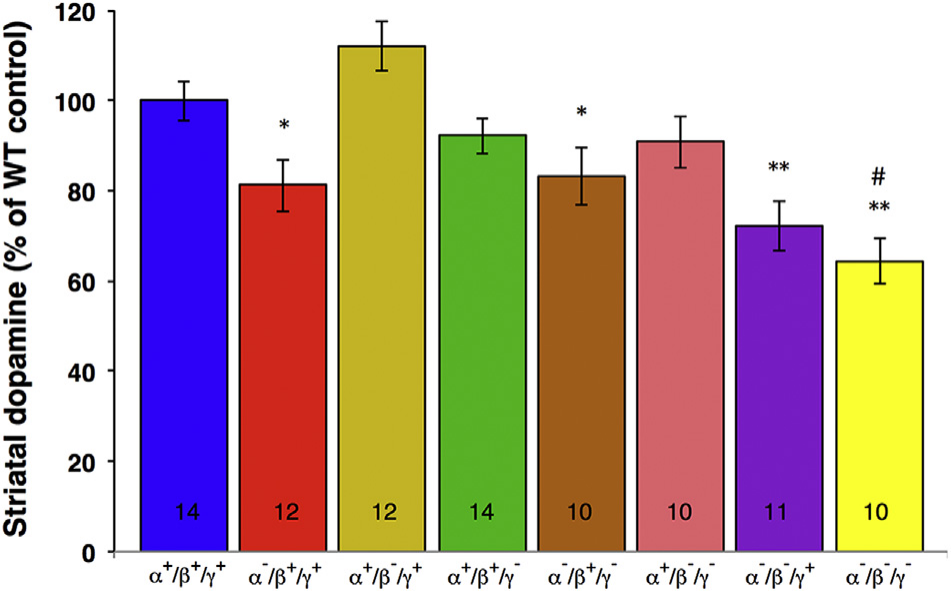
Dopamine levels in the dorsal striatum of 2-year-old synuclein knockout mice. Dopamine concentrations (in nanograms per milligram of protein) were normalized to the mean value for wild-type animals (100%). The bar chart shows means ± standard error of the mean data obtained from the number of samples shown at the bottom of the corresponding bar. A statistically significant difference between knockout and wild-type groups is denoted by asterisks (^∗^*p* < 0.05, ^∗∗^*p* < 0.01, Kruskal–Wallis analysis of variance with post hoc Mann–Whitney test) and between α-synuclein and α/β/γ-synuclein knockout groups–by hash (^#^*p* < 0.05).

To test if attenuated motor performance of aged synuclein-deficient mice correlates with changes in the nigrostriatal dopaminergic system, levels of dopamine and its major metabolites, DOPAC, and HVA were measured in extracts of the dorsal striatum of 2-year-old mice using HPLC with electrochemical detection. A statistically significant reduction in the striatal dopamine level compared with its level in the aged wild-type animals was observed in all groups of aged mice lacking α-synuclein, but this level was unaffected in aged animals lacking other members of the family as long as α-synuclein remained present (Fig. 4). Moreover, there was no statistically significant difference in the striatal dopamine levels between mice that lacked only α-synuclein and those additionally lacking either β- or γ-synuclein. However, deletion of all 3 synucleins did lead to a further reduction (Fig. 4). No statistically significant difference in levels of DOPAC and HVA was found between the studied groups, but the ratios of metabolites to dopamine, which are indicators of dopamine turnover, were significantly increased in aged mice lacking all 3 synucleins. In contrast, DOPAC to dopamine ratio was decreased in the striatum of mice lacking α- and β-synuclein (Table 1).

### 3.4 No age-dependent loss of dopaminergic neurons in the SNpc of synuclein-deficient mice

**Fig. 5:**
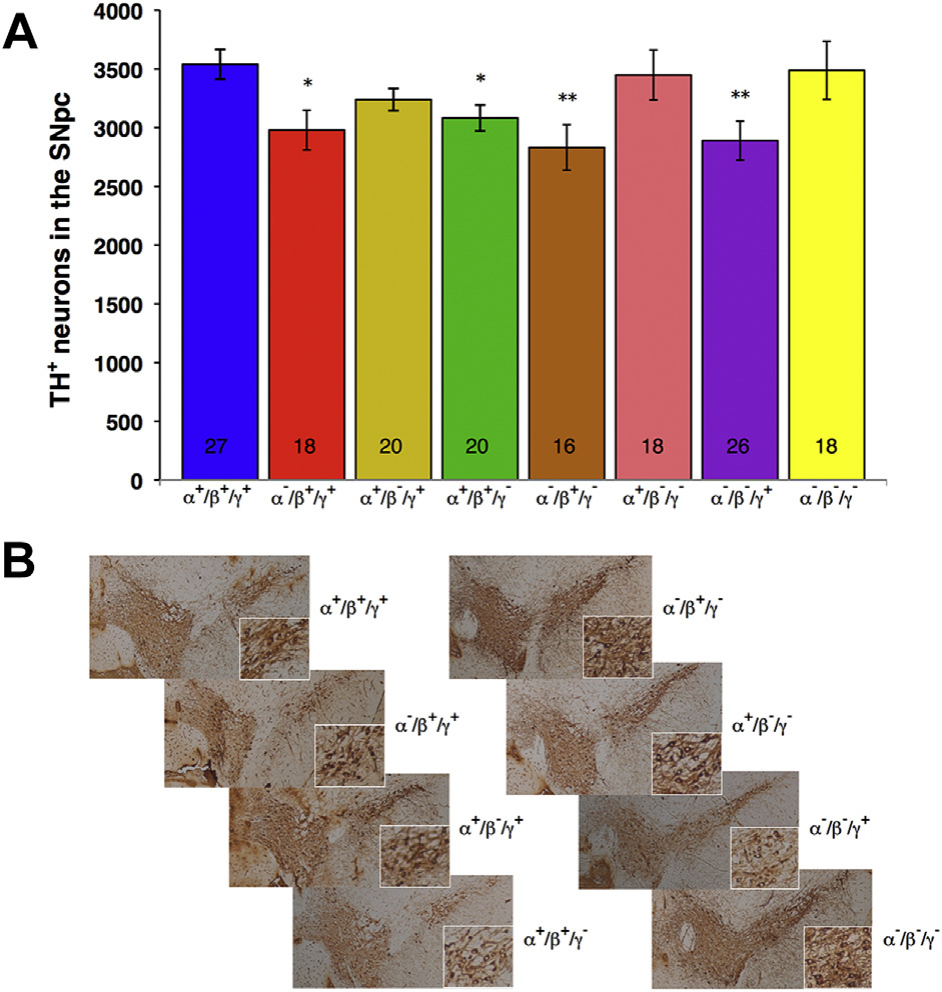
The number of dopaminergic neurons in the substantia nigra pars compacta (SNpc) of 2-year-old wild-type and various synuclein knockout mice. (A) The bar chart shows means ± standard error of the mean of stereologically counted TH-positive neurons per hemisphere. Asterisks denote statistically significant difference between knockout and wild-type groups (^∗^*p* < 0.05, ^∗∗^*p* < 0.01, Kruskal–Wallis analysis of variance with post hoc Mann–Whitney test). The number of samples processed for each group is shown at the bottom of the corresponding bar. (B) Representative images of anti-TH immunostained sections through the midbrain of the wild-type and synuclein deficient mice at the level between Bregma −3.0 to −3.2, where the border between SNpc and ventral tegmental area is well defined. Insets show SNpc dopaminergic neurons at higher magnification.

The number of TH-positive neurons was stereologically counted in the SNpc of aged synuclein-deficient mice. Consistent with previously reported data, small but significant reductions in number of these neurons were observed in single α-synuclein, single γ-synuclein, and α-synuclein/γ-synuclein double null mutant mice when compared with 2-year-old wild-type mice (Fig. 5), but degrees of these reductions were not greater than those in young adult mice of the same genotypes, suggesting that there is no further loss of dopaminergic neurons in aging synuclein-deficient mice. Similar decreased number of neurons was found in mice deficient for α- and β-synuclein, but the number of TH-positive neurons in the substantia nigra of 2-year-old triple synuclein null mutant mice was the same as that in age-matched wild-type mice (Fig. 5).

We did not reveal statistically significant correlation between the deficiency of SNpc dopaminergic neurons and the late-onset decrease of striatal dopamine content or animal performance in the accelerated rotarod test. The absolute value for Spearman's rank correlation coefficient (rho) was less than 0.5 for all groups with exception of the group of beta-synuclein null mutant animals, where the rho value was 0.6 for number of cells versus dopamine content, but this correlation was still not statistically significant (*p* > 0.05).

## 4 Discussion

In the present study, cohorts of aged wild-type mice and null mutant mice bearing inactivating mutations of the synuclein genes, all sharing the same C57Bl6 genetic background were produced within a single breeding programme, and several experimental paradigms were used to assess the role of synucleins in maintenance of the normal nigrostriatal system function in the aging brain. This approach allowed minimizing possible effects of indeterminate genetic and environmental factors when mice expressing different combinations of endogenous synucleins were compared. Several conclusions could be drawn from the results of these experiments.

First, we explicitly demonstrated that at least in the undemanding laboratory environment, mice do not require synucleins for executing major physiological processes even at an old age. Animals of all single and double synuclein null mutant genotypes were born at the expected Mendelian frequencies, were fertile, did not display any obvious morphologic or behavioral abnormalities, and no link was found between the absence of synuclein(s) and the life span of animals. These observations are consistent with previous reports from several laboratories that studied single or double synuclein null mutant mice (Abeliovich et al., 2000, Anwar et al., 2011, Cabin et al., 2002, Chandra et al., 2004, Dauer et al., 2002, Ninkina et al., 2003, Robertson et al., 2004, Schluter et al., 2003). Moreover, although triple synuclein null mutant mice maintained on a mixed genetic background developed clearly evident neurological phenotype (e.g., pronounced clasping reflex) at old age and had significantly reduced average life span (Burre et al., 2010), in our cohorts maintained on C57Bl6 background, triple synuclein null mutant mice had the same life span as wild-type mice and displayed no readily recognizable signs of neurological problems even at the age of 2 years. This suggests that certain unidentified factors can cooperate with the synuclein deficiency in triggering pathologic changes in the aging nervous system. Consequently, synucleins can be considered as factors increasing the robustness of animals and in particular, their nervous systems, to various challenges they might face in old age.

This notion is supported by subtle (subthreshold for causing changes in general animal physiology) but statistically significant changes of certain parameters revealed in our experiments focused on the nigrostriatal system in aging mice. The loss of dopaminergic neurons was observed in the SNpc of α-, γ-, α/γ-, and α/β-synuclein null mutant mice, but it was not progressive because the reduction in number of these neurons found in aged animals was not greater than the reduction seen in young animals of the same genotype (Garcia-Reitboeck et al., 2013, Robertson et al., 2004), indicating its developmental origin. Interestingly, animals lacking all 3 synuclein retain full complement of dopaminergic neurons in their SNpc. To explain this phenomenon, molecular mechanisms should be revealed that switch on during development of dopaminergic neurons only in response to complete absence of synucleins. It is feasible that activation of these mechanisms secures efficient survival of SNpc neurons during critical developmental periods.

A significant, ∼20%–30%, dopamine loss was found in the striatum of 2-year-old mutant mice lacking α-synuclein either singularly or in combination with other synucleins (i.e., in α-, α/γ-, α/β-, and α/β/γ-synuclein null mutant mice) but not in mice expressing this protein, independently of the presence of other synucleins. Moreover, additional depletion of other members of the family, either β- or γ-synuclein, did not have an additive effect on the loss of striatal dopamine seen in α-synuclein depleted brains, whereas depletion of all 3 family members produced such effect. These observations indicate that only α-synuclein is vital for maintaining normal dopamine metabolism in the nigrostriatal system of aged mice, whereas other 2 synucleins are also involved in this process, but their influence is marginal. Results obtained in previously published studies are consistent with these conclusions. No statistically significant loss of dopamine has been detected in the striatum of young adult (4 to 6-month old) α-, γ-, and α/γ-synuclein null mutant mice (Robertson et al., 2004). Therefore, striatal dopamine deficiency in α- and α/γ-synuclein null mutant mice observed in the present study is an age-related phenotype. However, in the striatum of young adult α/β- and α/β/γ-synuclein null mutant mice, dopamine loss comparable to that observed in aged mice has been reported (Anwar et al., 2011, Chandra et al., 2004). Because even aged β/γ-synuclein null mutant mice do not display decreased striatal dopamine content, it is feasible to suggest that α-synuclein is the most efficient of 3 family members in maintaining normal level of this neurotransmitter, β-synuclein is less efficient as it is able to compensate for α-synuclein loss in young but not in aged animals, and γ-synuclein does not play significant role in maintaining striatal dopamine level.

An incomplete overlap between synuclein null mutations causing the loss of dopaminergic neurons and causing striatal dopamine depletion as well as the absence of statistically significant correlation between these 2 parameters suggests an independent nature of these events in the brain of synuclein-deficient mice.

The increased metabolites to dopamine ratios (DOPAC:dopamine and HVA:dopamine) detected only in triple synuclein null mutant mice might imply that certain mechanisms compensating for reduced striatal dopamine levels by increasing turnover of this neurotransmitter become activated only when the interfamily functional redundancy cannot be employed.

The observed degree of striatal dopamine depletion was too small to cause a motor phenotype, which is commonly associated with the drop of dopamine content below 30% of the level in the striatum of healthy animals. However, we found that balance and coordination was compromised in several lines of aged synuclein null mutant mice, although this set of genotypes did not fully overlap with either a set displaying deficiency in the number of SNpc dopamine neurons or a set with depleted striatal dopamine content. A substantial decline of both inverted grid and rotarod performance was observed for all groups of animals lacking β-synuclein, that is, β-, β/γ-, α/β-, and α/β/γ-synuclein null mutant mice and once again, depletion of other synucleins had no additive effect. This suggests that the effect of β-synuclein deficiency on the motor performance of aged animals cannot be compensated by other members of the family. Because β-synuclein is highly expressed in many populations of brain neurons, it cannot be ruled out that compromised balance and coordination of animals lacking this protein is due to an improper function of not only their nigrostriatal system but also other neuronal systems involved in regulation of motor functions.

A functional redundancy within the synuclein family has been suggested based on a high degree of similarity between the family members. However, results of our studies suggest that each synuclein has a distinct set of functions, these 3 sets do not completely overlap and therefore, the loss of only some functions can be compensated by other family members, whereas the loss of other function can be aggravated by the absence of other family members. Still sufficient in a fully adept nervous system, this incomplete compensation can cause functional deficiencies in combination with certain other events that on their own have no or only subtle detrimental effect on the nervous system function. As a gradual accumulation of such subtle functional defects is a common trait in the aging nervous system, an impact of the loss of a synuclein function becomes particularly detrimental for aged animals. This might be relevant to certain neurodegenerative conditions characterized by the depletion of a functional pool of particular synuclein due to its intensive aggregation and trapping in pathologic inclusions.

## Disclosure statement

The authors declare no conflicts of interest.

## Figures and Tables

**Table 1:** Ratios of metabolites to dopamine contents in the striatum of synuclein null mutant mice

Genotype	α^+^/β^+^/γ^+^	α^−^/β^+^/γ^+^	α^+^/β^−^/γ^+^	α^+^/β^+^/γ^−^	α^−^/β^+^/γ^−^	α^+^/β^−^/γ^−^	α^−^/β^−^/γ^+^	α^−^/β^−^/γ^−^
DOPAC:DA								
Average	0.1019	0.1044	0.0940	0.0981	0.1150	0.0893	0.0807^∗^	0.1608^∗∗^
SEM	0.0062	0.0046	0.0047	0.0072	0.0043	0.0051	0.0053	0.0091
HVA:DA								
Average	0.0827	0.0814	0.0908	0.0832	0.0969	0.0911	0.0980	0.1090^∗^
SEM	0.0136	0.0059	0.0033	0.0059	0.0058	0.0080	0.0060	0.0026

*p < 0.05 and **p < 0.01 between knockout and wild-type groups, Mann–Whitney test.

Key: DA, dopamine; ​DOPAC, 3,4-dihydroxyphenylacetic acid; HVA, homovanillic acid; SEM, standard error of the mean.
